# Is the cross-over effect of a unilateral high-intensity leg extension influenced by the sex of the participants?

**DOI:** 10.1186/s13293-018-0188-4

**Published:** 2018-06-28

**Authors:** Aude-Clémence M. Doix, Felix Wachholz, Natalie Marterer, Lorenz Immler, Kathrin Insam, Peter A. Federolf

**Affiliations:** Department of Sport Science, University of Innsbruck, Fürstenweg 185, 6020 Innsbruck, Austria

**Keywords:** Cross-over effect, Motor irradiation, Force production, Electromyography, Maximum voluntary contraction MVC

## Abstract

**Background:**

While performing a unilateral muscle contraction, electrical muscle activity also arises in the contralateral homologous muscle, muscle group, or limb. When the muscle contraction induces muscle fatigue, females show not only a greater resistance than males but also a reduced contralateral muscle activation. The study aimed at investigating whether, during a high-intensity 30-s unilateral maximal effort isometric leg extension exercise, the contralateral non-exercising limb (NEL) knee extensor muscle activation would differ between females and males.

**Methods:**

Twenty participants, 11 females (23.80 ± 2.15 years old) and 9 males (26.50 ± 2.45 years old), performed a unilateral 30-s exercise while surface electromyography (sEMG) was measured from the vastus lateralis (VL), vastus medialis (VM), and rectus femoris (RF) on both limbs. The maximal voluntary contraction (MVC) was measured for both the exercising limb (EL) and the NEL before (MVC PRE) and after (MVC POST) the 30-s exercise to assess muscle fatigue.

**Results:**

While both females and males exhibited muscle fatigue in the EL (*p* = 0.015), females exhibited a lower MVC reduction than males (*p* = 0.042), suggesting that females were less fatigued than males. Although no muscle fatigue, i.e., no MVC force reduction was found in the NEL for either group before and after the 30-s exercise, the muscle activity of the VL was found to be of greater magnitude during the MVC POST only for females (*p* = 0.047) while it remained unchanged for males. During the 30-s exercise, the force output of the EL decreased only for males (*p* = 0.029) while females showed a preservation of the force output (*p* > 0.05). The sEMG activity of the NEL during the 30-s unilateral exercise increased for both groups in all measured muscles (all *p*-values < 0.03).

**Conclusions:**

Likely, different underlying muscle fatigue mechanisms occurred in the EL between females and males. Yet, our findings suggest that the cross-over effect to the NEL during the 30-s exercise occurred in a similar fashion in both groups. The current study suggests that the contralateral muscle activation seen with a unilateral exercise is independent of the sex of individuals. Therefore, unilateral training or rehabilitation-based protocols would similarly impact females and males.

## Background

Following neurological disease, such as stroke, cerebral palsy, brain injury, or periods of limb immobilization after an accident or an injury, some rehabilitation protocols are performed unilaterally to restore as much as possible the bilateral motor function [1]. Unilateral muscle contraction not only generates activity in the targeted muscle group but also irradiates within the contralateral homologous muscles [2]. This involuntary irradiation of muscle activity has been shown during intermittent, sustained, submaximal, and maximal muscle contractions [3, 4], whether muscle fatigue arises or not. In literature, this phenomenon is termed cross-over, or cross-transfer effect, motor irradiation or non-local muscle fatigue [2, 4–6].

Muscle fatigue is expressed as the reduction in maximal voluntary force production [7–9] and encompasses both peripheral and central components. While the first refers to the impaired muscle capacity to generate force within the muscle fiber, modifications in the voluntary neural drive from the corticospinal pathway innervating muscles may concurrently occur [9, 10].

The cross-over effect of unilateral muscle fatigue depends on the muscle group or task [5, 11–14]. After a 100-s unilateral sustained maximal voluntary contraction (MVC) of knee extensors, the cross-over effect of muscle fatigue in the resting contralateral limb was observed to be of greater magnitude in males compared to females [13]. Another study showed that the plantar flexor MVC was reduced after bilateral sustained handgrip contractions [12]. Also, Halperin and colleagues [11] showed that while non-exercised knee extensors exhibited a cross-over effect of fatigue after both unilateral knee extensors and elbow flexors, this was not observed to occur in the contralateral elbow flexors. Central mechanisms both at supraspinal [15–18] and spinal levels [15, 19–23] have been demonstrated to play a role in this limb interaction [24]. Mostly, the reduction in MVC in the non-exercising contralateral limb was found to be accompanied by a diminution in voluntary activation in the knee extensors [5, 11, 13, 25], ankle plantar flexors [12, 22], and first dorsal interossei [26]. The cross-over effect could occur to balance bilateral activity for two-limb coordinated and automatic tasks (e.g., balance or locomotion) and more generally to maintain lower limbs homeostasis [5].

It is now well established that because of physiological and structural differences, the development of muscle fatigue differs between sexes [27, 28], females being more fatigue-resistant than males [28]. The underlying mechanisms may arise from different sources both at peripheral and central levels of the motor pathway. It is likely that the greater proportion of type I muscle fibers with greater oxidative capacity play a role in the lower fatigability of females [29, 30]. Additionally, with greater muscle size, the contraction force produced is higher leading to a greater compression of the blood vessels of the active muscle in males. Thus, this reduces the blood perfusion and increases the accumulation of by-products in males compared to females [27]. Small diameter groups III and IV muscle afferents provide information regarding the strain and the metabolic milieu modifications in the muscle [31–35]. The groups III and IV afferents have been shown to act as a negative feedback loop at the central levels, onto the α-MNs pool [36, 37] and are also likely increase the pre-synaptic inhibition [38, 39]. Therefore, with lower muscle mass in females, both the muscle perfusion is higher and the intramuscular blood flow pressure is lower than in males, reducing the role of muscle afferents onto the females’ muscle performance [28, 40]. Finally, another potential mechanism is attributed to a difference in muscle activation, as females seem more likely to exhibit a greater preservation of voluntary activation than males [13, 27, 28, 41].

A few studies have investigated the influence of the sex of individuals knee extensors muscle fatigue both during isometric sustained submaximal [42–45] and maximal [13, 46, 47] levels of muscle contractions. Yet, the effect of sex of a unilateral sustained muscle contraction to the contralateral muscle group has been scarcely researched. To our knowledge, only two studies approached this question [13, 47]. First, Martin and Rattey [13] showed that after a 100-s sustained isometric knee extension MVC, females were found to be more fatigue-resistant than males with a lower magnitude of central fatigue resulting in a lower magnitude of the cross-over effect than males [13]. These differences between females and males appeared after 40 s of sustained effort [13]. Second, Ye and colleagues [47], using a fatiguing protocol of six sets of unilateral 30-s isometric knee extension MVCs, showed that females did not exhibit a cross-over effect on the contralateral knee extensors, while males did. It is to be noted that one study [47] reported small effect sizes (for females: Cohen’s *d* = 0.07; for males: Cohen’s *d* = 0.13), while the other one [13] did not include effect sizes. The magnitude of the cross-over effect is multifaceted and depends on the muscle group, task, sex, or experimental protocol [5, 11–14, 47]. When investigating the influence of the sex of the participants, Martin and Rattey [13] and Ye and colleagues [47] used long-duration sustained isometric fatiguing contractions respectively of 100 s and six sets of 30 s. Thus, it seems appropriate to reexamine the question of sex differences in cross-transfer phenomena with varied experimental procedures, for example, with a short-duration exercise. Two important factors of shorter duration exercise can be mentioned. First, after short-duration and high-intensity efforts, typically like a 30-s MVC, the ATP-PC (adenosine triphosphate-phosphocreatine) within the muscle is depleted and the build-up of H^+^ (hydrogen) ions and Pi (inorganic phosphate) for instance triggers the small group muscle afferents to discharge modifying the neural drive towards the active muscle [31–36, 38, 39]. Second, 100 s [13] or six times 30 s [47] are strenuous protocols and potentially uncomfortable for health-compromised population in clinical settings. Therefore, the use of a shorter duration can be of interest for the comfort of patients.

Therefore, the aim of this study was to investigate whether the sex of the participants would influence the activation of the knee extensor muscles in the contralateral non-exercising limb during a short-duration unilateral leg extension during a sustained MVC of 30 s. We hypothesized that the sex of the participants would not influence the contralateral muscle activity. In both males and females, it was expected that a cross-over effect would be seen in the contralateral limb resulting in a continuous increase in the electromyogram (EMG) of the non-exercising knee extensors during the exercise.

## Methods

### Participants

Twenty-one young healthy participants took part in the present study. However, one participant developed knee pain during the experimental procedure and was therefore excluded from the analysis. Thus, data from 20 participants (Table 1) were analyzed. The participants were sport sciences faculty students and were recreationally active and self-reported their weekly hours of participation in physical activity. Participants did not report any pathology and neurological complications or muscular, tendon, or joint injury within 6 months prior to the experiment.

**Table 1:** Participants characteristics. Mean (and standard deviation) of participants characteristics

	Age (years)	Height (cm)	Weight (kg)	Physical activity (h/week)
Females, *N* = 11	23.80 (2.15)	170.40 (6.02)	62 (4.24)	9.5 (4.5)
Males, *N* = 9	26.50 (2.45)	183.38 (6.72)	79.50 (4.78)	16.6 (8.5)

### Study design

**Fig. 1:**
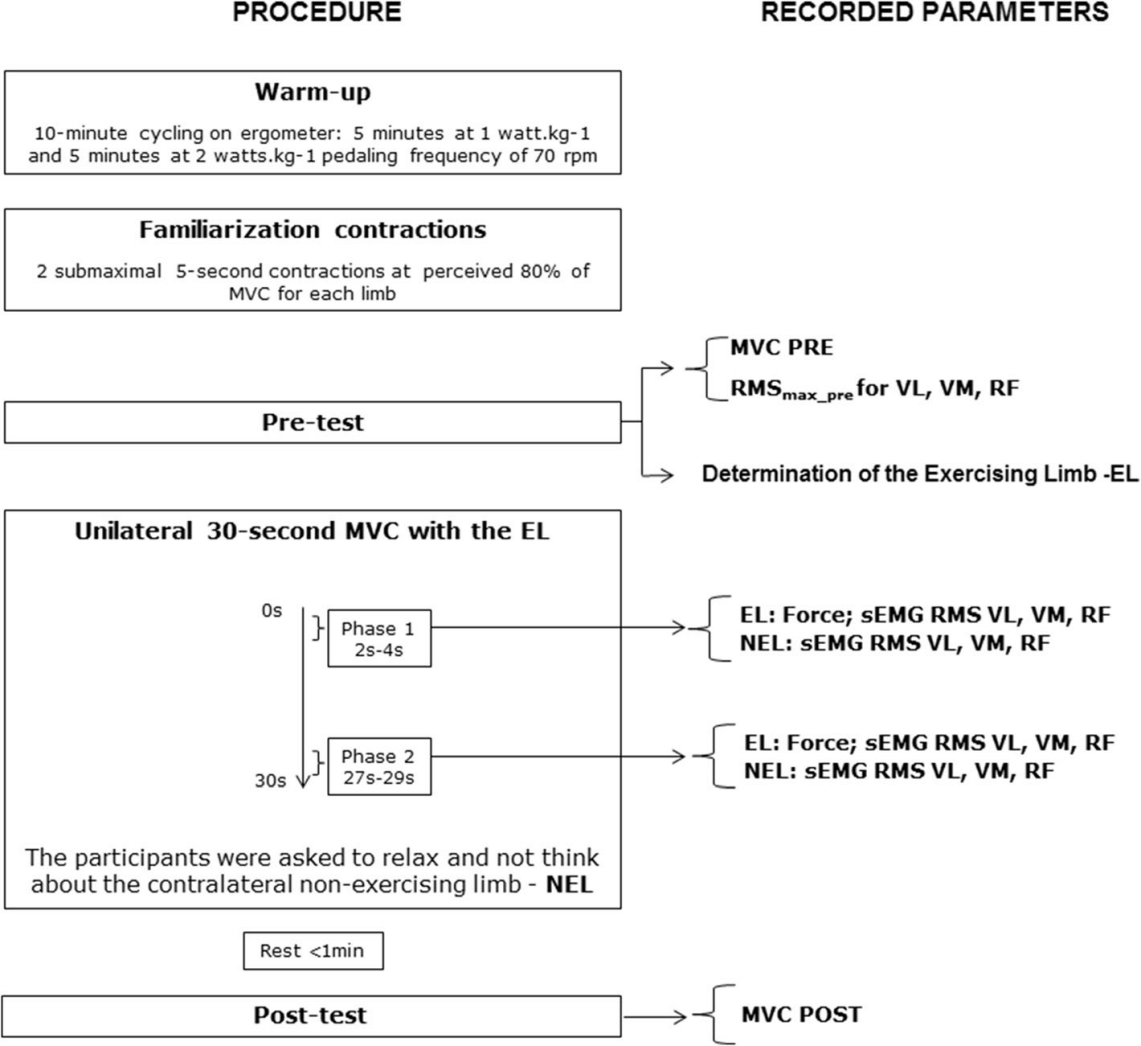
Graphical representation of the experimental protocol and the variables used for data analysis. MVC, maximal voluntary contraction; before (PRE) and after (POST) the 30-s unilateral isometric maximal leg extension exercise; RMS_max_pre_, root mean square maximal value recorded during the best MVC PRE; VL, vastus lateralis; VM, vastus medialis; RF, rectus femoris; EL, exercising limb; NEL, non-exercising limb

As illustrated in Fig. 1, the participants performed a 10-min warm-up on a cycling ergometer (Daum Electronics, Ergo Bike, 8008 TRS, Fürth, Germany) with the first 5 min at 1 W kg^−1^ and the last 5 min at 2 W kg^−1^ at a pedaling frequency of 70 rpm. After warming up, the participants were transferred and secured to a force measurement device. To quickly habituate the participant with the testing, they were asked to perform two 5-s bouts of unilateral submaximal muscle contractions on both legs, at what would correspond to 80% of their perceived maximal force production.

The measurements started with two 5-s MVCs per limb, which were alternated between the left and right leg with 5 s of rest (MVCs PRE), starting always with the right leg. The strongest limb, i.e., the limb having produced the greatest force output, was then chosen to be the exercising limb (EL). The highest MVC of the two repetitions per limb was considered for further analysis.

Subsequently, a 30-s unilateral isometric MVC leg extension exercise was conducted with the EL. The participants were informed of the duration of the exercise and were instructed to push as hard as possible (all-out effort) during the entire test. Participants were instructed to relax and not think about the non-exercising limb (NEL), but they were not aware that the goal of the study was to assess the NEL. No visual feedback was provided to the participants. During the exercise, the participants were strongly verbally encouraged by the experimenters.

After the unilateral 30-s leg extension exercise and a subsequent resting period (less than a minute), two additional 5-s MVCs per limb (MVC POST) were recorded; the EL was always measured first. In both PRE and POST MVCs, the larger force or muscle activation values of the two repetitions were selected for further analysis.

### Equipment setup

#### Force measurements

Force production of the leg extension muscles was measured in a custom-made device with the tested leg in a closed kinetic chain [48]. A force transducer (model Wägezelle HMB Z7AD1, linear range 0–5 kN, precision ± 0.5%, sensitivity 1.2 mV/V; Hottinger Baldwin Messtechnik, Darmstadt, Germany) measured force production on the footplate. During all contractions, participants were seated with the footplate and the seat adjusted at the same height. The hip and ankle angles were adjusted to 90°, and the knee angle was set to 120° (180° being the full knee extension). All joint angles were manually controlled using a mechanical goniometer. The foot of the EL was firmly tightened with straps onto the footplate. The NEL was not fixed and left hanging in the aperture of the apparatus. While the thigh was resting on the seat, the lower leg had no contacts with the device nor the ground. Participants were asked to relax their leg. Upper body movements of the participants were constrained by two cross-over shoulder belts connected to each other over the abdomen. Participants were allowed to grip side-mounted handles during the testing procedure. The load cell signal was sampled at 1500 Hz and acquired directly to a custom-built recording program, in LabView (LabVIEW, National Instruments, Austin, TX, USA). The reliability of this test bench was reported with an ICC test-retest value of 0.95 for females and 0.96 for males [49].

#### Surface electromyography (sEMG recordings)

Bipolar sEMG electrodes with conductive wet gel (Ag/AgCl^−^, recording diameter of 1 cm, 2 cm inter-electrode distance, Ambu® BlueSensor P Ambu A/S, Ballerup, Denmark) were positioned over the vastus lateralis (VL), the vastus medialis (VM), and the rectus femoris (RF) on both limbs, according to the SENIAM recommendations [50]. The reference electrode was placed on the medial malleolus of the left ankle. The low-resistance impedance between electrodes (< 5 kΩ) was obtained after shaving and cleaning the skin with alcohol. A Noraxon TeleMyo™ 2400T G2 Direct Transmission system (Noraxon Inc., Scottsdale, AZ, USA) with wireless bipolar surface EMG sensors was used to record sEMG data at a sampling rate of 1500 Hz. The sEMG signals were amplified (gain = 500) and bandpass-filtered from 10 to 500 Hz (common mode rejection ratio = 100 dB). Force and sEMG signals were synchronized and simultaneously collected by the Noraxon system.

### Data analysis

#### Maximal voluntary contractions

The MVC was considered as the mean value over a 500-ms period around the force output peak. In PRE tests, the best MVC of the two trials was analyzed.

#### EMG analysis

The root mean square (RMS) of the VL, VM, and RF muscle electromyographic signals was calculated (Noraxon Inc., Scottsdale, AZ, USA). The maximal sEMG RMS activation was determined as the mean value over the same 500-ms period around the force peak. In the PRE and POST tests, the MVC trial with the highest force peak was analyzed. During the 30-s unilateral exercise, the sEMG RMS values of the three muscles were analyzed as averages over two 2-s phases. Phase 1 comprised the 2nd to 4th second into the exercise and phase 2 the 27th to 29th second. All RMS values obtained during phase 1 and phase 2 were normalized to their maximal value obtained for that muscle during the MVC PRE (RMS/RMSmax_pre). The EMG RMS values obtained during the MVC PRE and the MVC POST were also normalized their maximal value obtained for that muscle during the MVC PRE (RMSmax_post/RMSmax_pre). For illustrative purposes only, the sEMG RMS signal was filtered with a fourth-order low-pass Butterworth filter and was then zero-phase filtered.

#### Force output during the sustained 30-s exercise

In a similar fashion as the sEMG analysis, two 2-s phases where used and averaged to analyze the force decrement from phase 1 to phase 2. In addition, the slope of the force profile output was calculated with a first-degree polynomial to compute the force output change.

### Statistical analysis

Statistical processing was performed in PASW Statistics version 24 (SPSS Inc., Chicago, IL, USA). The Shapiro-Wilk test was used to test whether outcome measures were normally distributed. In all statistical analyses, the significance level was set to *α* = 0.05, and all data are expressed as means ± SE (standard error of the mean) in the entire manuscript and in tables and figures.

When data did not follow a normal distribution, a logarithmic transformation was computed to test the data with a two-way analysis of variance (two-way ANOVA). The MVCs and EMG RMS activity measured in PRE and POST tests were analyzed with a two-way ANOVA with repeated measures to test for main effects of time (PRE and POST) and sex, as well as interactions time × sex. When a main and/or an interaction effect was found, pairwise comparison with a Bonferroni correction post hoc analysis was applied. Effect size from partial eta^2^ values (*η*^2^_*p*_) and the observed power were computed.

For the 30-s exercise, the force output of the EL and the sEMG of both limbs were analyzed with a two-way ANOVA with repeated measures to test for main effects of time (phases 1 and 2) and sex, as well as interactions time × sex. When a main and/or an interaction effect was found, pairwise comparison with a Bonferroni correction post hoc analysis was used. Finally, the slope of the force output profile was analyzed with a *t* test.

## Results

### Maximal voluntary contractions

**Fig. 2:**
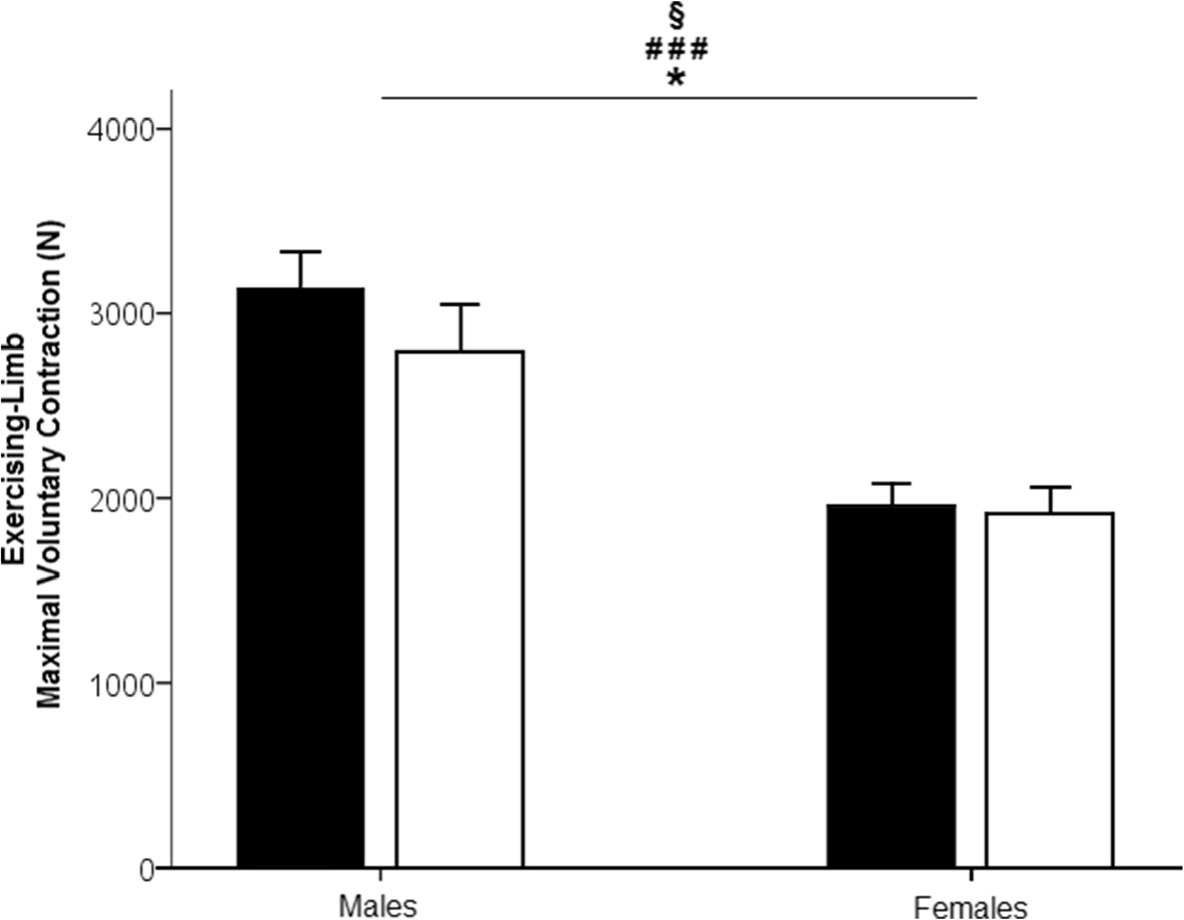
Maximal voluntary isometric force of the exercising limb (EL) in Newtons (N) for the male group and the female group, before (in black) and after (in white) the 30-s unilateral exercise. Bars represent group mean values. Error bars are the standard error of the mean. Significant main time differences *p* < 0.05 (*), time × sex interaction *p* < 0.05 (§), main sex differences *p* < 0.001 (###)

Overall and as expected, the MVC of the EL significantly decreased from PRE to POST tests (main time effect: *F*_(1,18)_ = 7.227; *p* = 0.015; *η*^2^_*p*_ = 0.286; observed power = 0.720; Fig. 2). The participants’ sex affected this decline (interaction effect: *F*_(1,18)_ = 4.791; *p* = 0.042; *η*^2^_*p*_ = 0.210; observed power = 0.544) and also showed a main effect of sex (*F*_(1,18)_ = 17.859; *p* = 0.001; *η*^2^_*p*_ = 0.494; observed power = 0.977). Females had a lower MVC reduction than males (post hoc *p* < 0.001) with an average decrease of MVC for females of 2.04 ± 2.57%, whereas the decline was of 11.46 ± 5.12% for males.

The associated maximal sEMG RMS muscle activity (Table 2) of the EL did not modify from PRE to POST in the VL (*F*_(1,18)_ = 0.008; *p* = 0.928; *η*^2^_*p*_ = 0.000; observed power = 0.051), and neither any main effect of gender (*F*_(1,18)_ = 2.860; *p* = 0.108; *η*^2^_*p*_ = 0.137; observed power = 0.360) nor any interaction were found (*F*_(1,18)_ = 2.860; *p* = 0.108; *η*^2^_*p*_ = 0.137; observed power = 0.360). For the VM, the difference did not reach the significance level (main time effect: *F*_(1,18)_ = 4.060; *p* = 0.059; *η*^2^_*p*_ = 0.184; observed power = 0.479), and no interaction with the sex of the participants (*F*_(1,18)_ = 0.903; *p* = 0.354; *η*^2^_*p*_ = 0.048; observed power = 0.147) nor any main effect of sex was found (*F*_(1,18)_ = 0.903; *p* = 0.354; *η*^2^_*p*_ = 0.048; observed power = 0.147). Likewise for the RF, no overall time effect was found (*F*_(1,18)_ = 1.699; *p* = 0.209; *η*^2^_*p*_ = 0.086; observed power = 0.235), no interaction with the sex of the participants (*F*_(1,18)_ = 2.594; *p* = 0.125; *η*^2^_*p*_ = 0.126; observed power = 0.332), nor any main effect of sex were found (*F*_(1,18)_ = 2.594; *p* = 0.125; *η*^2^_*p*_ = 0.126; observed power = 0.332).

**Table 2:** Mean (and standard error of the mean) of the normalized maximal surface electromyographic root mean square (sEMG) expressed in percentage (%) values of the vastus lateralis, the vastus medialis, and the rectus femoris of the exercising limb and the non-exercising limb measured during the maximal voluntary contractions before (PRE) and after (POST) the 30-s unilateral exercise of the EL for both the females and males

	Vastus lateralis		Time × sex	Sex	Vastus medialis		Time × sex	Sex	Rectus femoris		Time × sex	Sex
	PRE	POST			PRE	POST			PRE	POST		
Exercising limb												
Males	100 (0.00)	107.07 (21.40)	*p* = 0.108	*p* = 0.108	100 (0.00)	97.59 (13.53)	*p* = 0.354	*p* = 0.354	100 (0.00)	101.28 (21.92)	*p* = 0.125	*p* = 0.125
Females	100 (0.00)	92.12 (18.18)			100 (0.00)	93.27 (6.08)			100 (0.00)	87.81 (15.46)		
	Time		*p* = 0.928				*p* = 0.059^£^				*p =* 0.209	
Non-exercising limb												
Males	100 (0.00)	86.85 (22.66)	*p = 0.047*	*p = 0.047*	100 (0.00)	98.39 (24.30)	*p* = 0.881	*p* = 0.881	100 (0.00)	93.24 (29.29)	*p* = 0.254	*p* = 0.254
Females	100 (0.00)	136.90 (80.62)			100 (0.00)	100.31 (30.55)			100 (0.00)	172.85 (244.51)		
	Time		*p* = 0.904				*p* = 0.919				*p =* 0.840	

Significant *p* values are in italics, and a strong trend is indicated with the ^£^ symbol

**Fig. 3:**
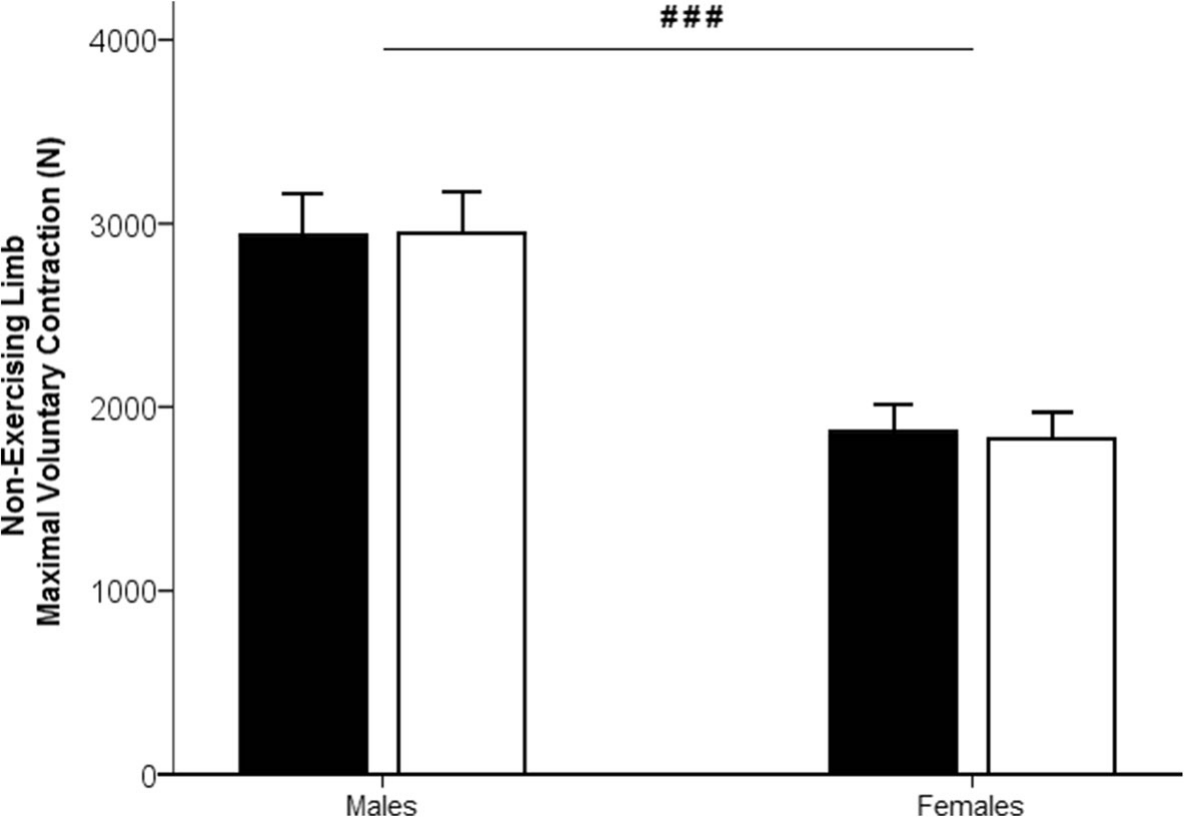
Maximal voluntary isometric force of the non-exercising limb (NEL) in Newtons (N) for the male group and the female group, before (in black) and after (in white) the 30-s unilateral exercise. Bars represent group mean values. Error bars are the standard error of the mean. Significant main sex differences *p* < 0.001 (###), main time differences, and time × sex interaction were not significant

No significant reduction of the NEL MVC (Fig. 3) was found after the 30-s exercise (main time effect: *F*_(1,18)_ = 0.084; *p* = 0.775; *η*^2^_*p*_ = 0.005; observed power = 0.059) nor any interaction (*F*_(1,18)_ = 0.562; *p* = 0.463; *η*^2^_*p*_ = 0.030; observed power =0.110). Yet, a main effect of sex was found (*F*_(1,18)_ = 18.341; *p* < 0.001; *η*^2^_*p*_ = 0.505; observed power = 0.981), with females generating less maximal force output than males from PRE to POST tests (post hoc: *p* < 0.001).

In the NEL (Table 2), the muscle activation of the VL overall did not change from PRE to POST (*F*_(1,18)_ = 0.015; *p* = 0.904; *η*^2^_*p*_ = 0.001; observed power = 0.052), but an interaction with sex (*F*_(1,18)_ = 4.562; *p* = 0.0.047; *η*^2^_*p*_ = 0.202; observed power = 0.525) and a main effect of sex were found (*F*_(1,18)_ = 4.562; *p* = 0.047; *η*^2^_*p*_ = 0.202; observed power = 0.525). Females exhibited an increase in the VL sEMG RMS activity while males exhibited a decrease in muscle activation. Yet, the VM and the RF, they did not modify over time (VM—main time effect: *F*_(1,18)_ = 0.011, *p* = 0.919, *η*^2^_*p*_ = 0.001, observed power = 0.051; RF—main time effect: *F*_(1,18)_ = 0.042; *p* = 0.840; *η*^2^_*p*_ = 0.002; observed power = 0.054) and neither any interaction with the sex of the participants (VM—*F*_(1,18)_ = 0.023; *p* = 0.881; *η*^2^_*p*_ = 0.001; observed power = 0.051; RF—*F*_(1,18)_ = 1.390; *p* = 0.254; *η*^2^_*p*_ = 0.072; observed power = 0.201) nor any main effect of the sex of the participants appeared for both the VM (*F*_(1,18)_ = 0.023; *p* = 0.881; *η*^2^_*p*_ = 0.001; observed power = 0.052) and the RF (*F*_(1,18)_ = 1.390; *p* = 0.254; *η*^2^_*p*_ = 0.072; observed power = 0.201).

### Thirty-second unilateral exercise

**Fig. 4:**
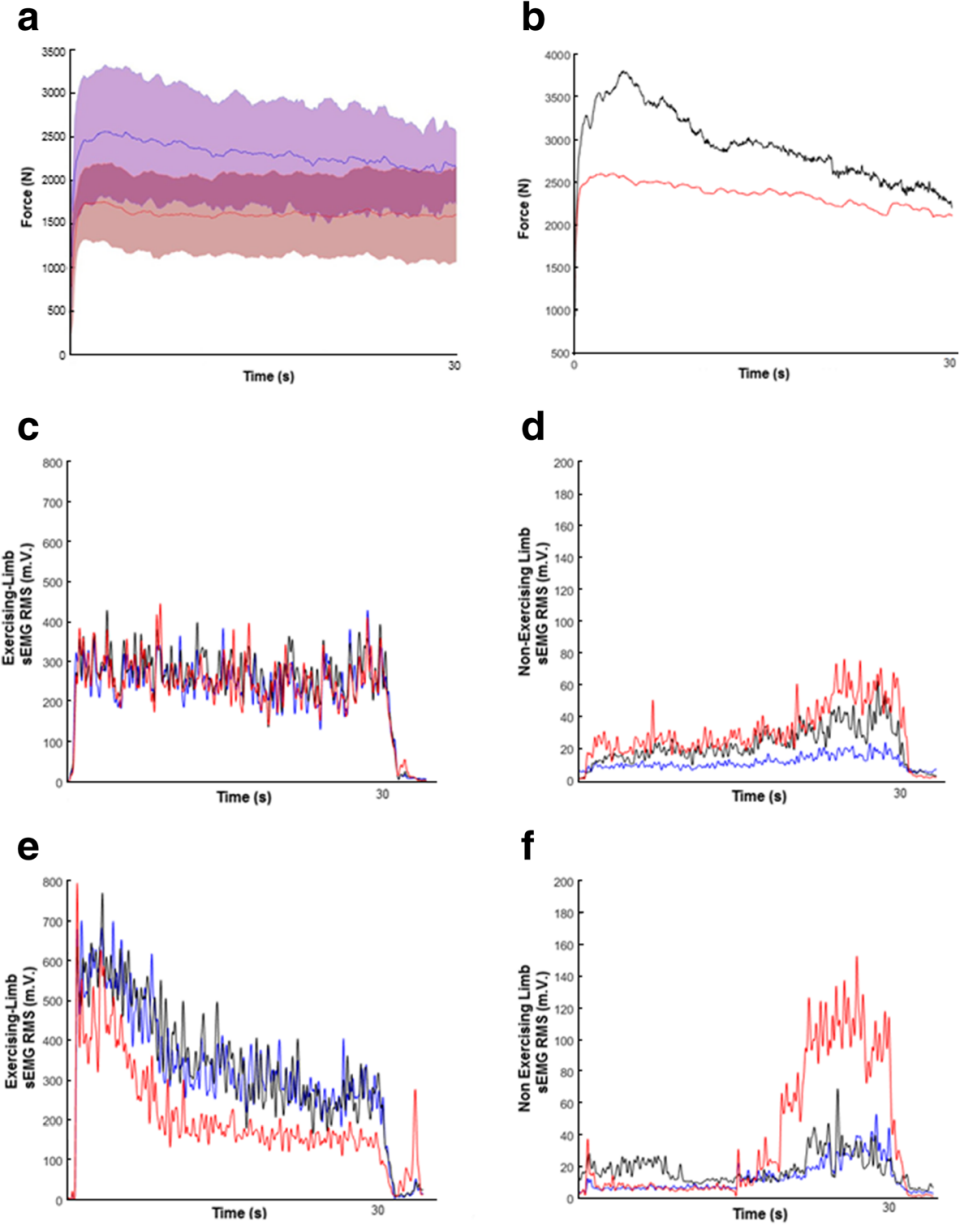
**a** Average (plain line) and standard deviation (shaded areas) of the force output in Newtons (N) during the 30-s unilateral exercise for the female group (average: red plain line; standard deviation: red shaded area) and for the male group (average: blue plain line; standard deviation: blue shaded area). **b** Representative recordings of the force output in Newtons (N) during the 30-s unilateral exercise for one male (black trace) and one female (red trace). **c**, **d** Representative recordings of the female participant’s surface electromyographic root mean square (sEMG RMS) of the exercising limb (EL—panel **c**) and the non-exercising limb (NEL—panel **d**), vastus lateralis (blue trace), vastus medialis (black trace), and rectus femoris (red trace) during the 30-s unilateral exercise. **e**, **f** Representative recordings of the male participant’s surface electromyographic root mean square (sEMG RMS) of the exercising limb (EL—panel **e**) and the non-exercising limb (NEL—panel **f**), vastus lateralis (blue trace), vastus medialis (black trace), and rectus femoris (red trace) during the 30-s unilateral exercise

Overall, during the 30-s unilateral exercise, the force production of the EL significantly diminished from phase 1 to phase 2 (*F*_(1,18)_ = 9.517; *p* = 0.006; *η*^2^_*p*_ = 0.346; observed power = 0.831), without any interaction with the sex of the participants (*F*_(1,18)_ = 2.446; *p* = 0.135; *η*^2^_*p*_ = 0.120; observed power = 0.316) nor any main effect of the sex (*F*_(1,18)_ = 0.386; *p* = 0.542; *η*^2^_*p*_ = 0.021; observed power = 0.091). Figure 4a (top panel) shows the mean and SD force output produced during the 30-s unilateral exercise for the female (red line) and male groups (blue line). Figure 4b illustrates one representative female (in red) and one representative male (in black). The female participant was capable of maintaining a rather constant force output whereas the male participant exhibited a constant decline in force production. In participants altogether, the force output profile slope was found to decrease significantly for males (*T* = − 2.654; *p* = 0.029; slope = − 14.06 ± 5.30) but not for females (*p* = 0.364; slope = − 2.71 ± 2.85).

The sEMG RMS of the muscle activity for both the EL and the NEL during the 30-s exercise is shown in Fig. 4 for one representative female (panels c and d) and one representative male (panels e and f). As seen in the EL (panels b and d), while the female participant (panel c) tended to maintain a somewhat constant muscles activation, the male exhibited a continuous decline throughout the exercise (panel e). Concomitantly, the muscle activation of the NEL increased for both the females (panel d) and the males (panel f).

**Fig. 5:**
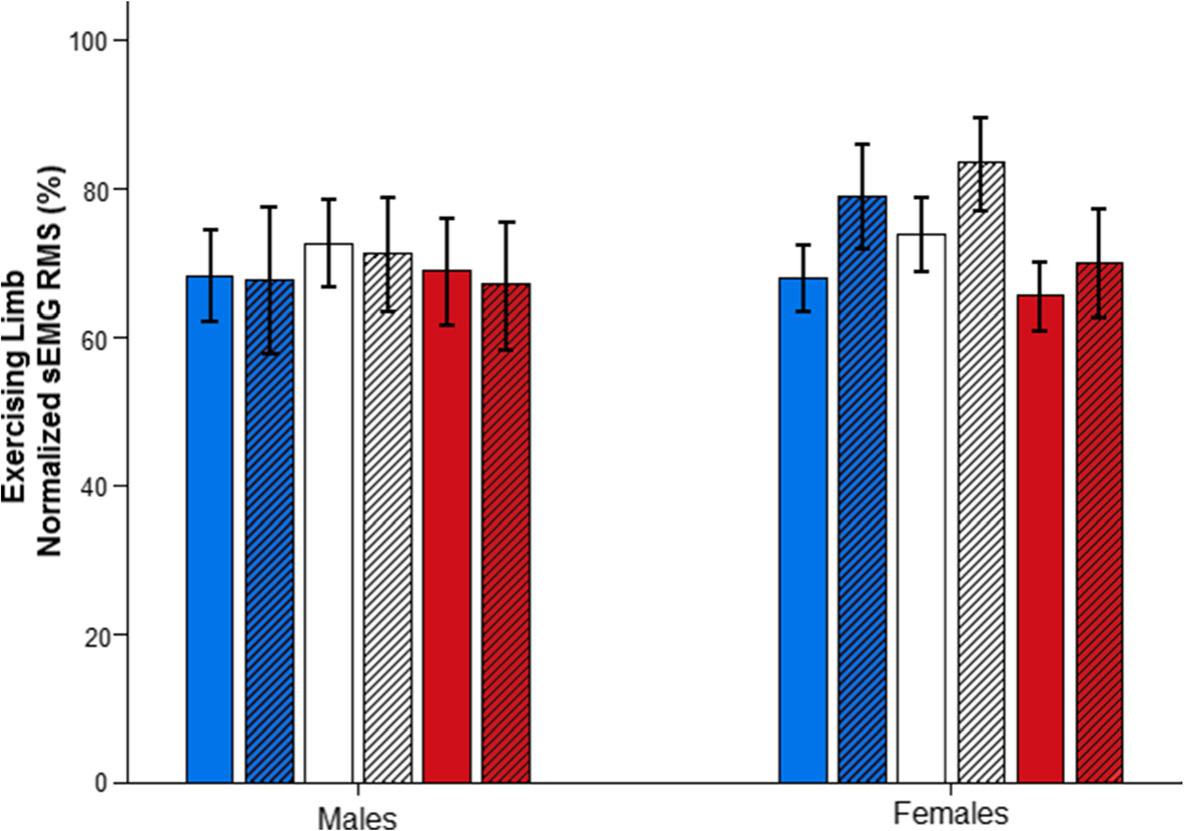
Normalized surface electromyographic (sEMG) values for both the male group and the female group of the vastus lateralis (blue), the vastus medialis (white), and the rectus femoris (red) of the exercising limb (EL) for phase 1 (plain fill-in) and phase 2 (dashed fill-in). Bars represent group mean values. Error bars are the standard error of the mean

The associated normalized sEMG RMS of the EL during the 30-s exercise (Fig. 5) did not modify for any of the muscles measured (VL—main time effect: *F*_(1,18)_ = 1.447; *p* = 0.245; *η*^2^_*p*_ = 0.074; observed power = 0.207; main sex effect: *F*_(1,18)_ = 0.386; *p* = 0.542; *η*^2^_*p*_ = 0.021; observed power = 0.091; interaction time × sex: *F*_(1,18)_ = 1.814; *p* = 0.195; *η*^2^_*p*_ = 0.092; observed power = 0.247; VM—main time effect: *F*_(1,18)_ = 0.866; *p* = 0.364; *η*^2^_*p*_ = 0.046; observed power = 0.143; main sex effect: *F*_(1,18)_ = 0.765; *p* = 0.393; *η*^2^_*p*_ = 0.041; observed power = 0.132; interaction time × sex: *F*_(1,18)_ = 1.567; *p* = 0.227; *η*^2^_*p*_ = 0.080; observed power = 0.220; RF—main time effect: *F*_(1,18)_ = 0.099; *p* = 0.756; *η*^2^_*p*_ = 0.005; observed power = 0.060; main sex effect: *F*_(1,18)_ = 0.000; *p* = 0.987; *η*^2^_*p*_ = 0.000; observed power = 0.105; interaction time × sex: *F*_(1,18)_ = 0.548; *p* = 0.469; *η*^2^_*p*_ = 0.030; observed power = 0.108).

**Fig. 6:**
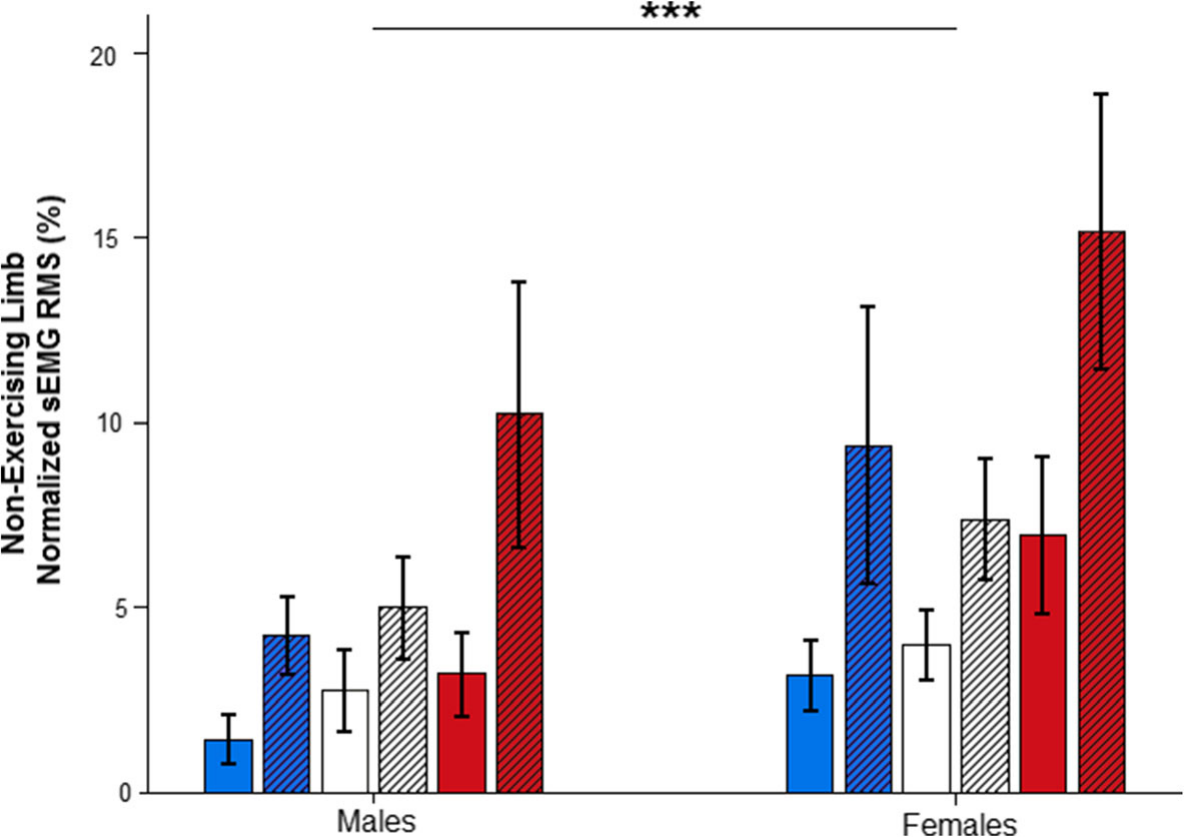
Normalized surface electromyographic (sEMG) values for both the male group and the female group of the vastus lateralis (blue), the vastus medialis (white), and the rectus femoris (red) of the non-exercising limb (EL) for phase 1 (plain fill-in) and phase 2 (dashed fill-in). Bars represent group mean values. Error bars are the standard error of the mean. Significant main time difference *p* < 0.001 (***)

However, the normalized sEMG RMS of the NEL (Fig. 6) was found to increase in all of the measured muscles without any main nor interaction with the sex of the participant effects (VL—main time effect: *F*_(1,18)_ = 35.766; *p* < 0.001; *η*^2^_*p*_ = 0.665; observed power = 1.000; interaction time × sex: *F*_(1,18)_ = 1.305; *p* = 0.268; *η*^2^_*p*_ = 0.068; observed power = 0.191; main sex effect: *F*_(1,18)_ = 3.223; *p* = 0.089; *η*^2^_*p*_ = 0.152; observed power = 0.397; VM—main time effect: *F*_(1,18)_ = 45.478; *p* < 0.001; *η*^2^_*p*_ = 0.716; observed power = 1.000; interaction time × sex: *F*_(1,18)_ = 0.348; *p* = 0.563; *η*^2^_*p*_ = 0.019; observed power = 0.086; main sex effect: *F*_(1,18)_ = 2.795; *p* = 0.112; *η*^2^_*p*_ = 0.134; observed power = 0.353; RF—main time effect: *F*_(1,18)_ = 33.837; *p* < 0.001; *η*^2^_*p*_ = 0.653; observed power = 1.000; interaction time × sex: *F*_(1,18)_ = 1.539; *p* = 0.231; *η*^2^_*p*_ = 0.079; observed power = 0.217; main sex effect: *F*_(1,18)_ = 2.819; *p* = 0.110; *η*^2^_*p*_ = 0.135; observed power = 0.356).

## Discussion

The main purpose of the current study was to investigate whether the sex of the participants affects the activation of the contralateral NEL knee extensors during a 30-s unilateral sustained MVC. The results suggest that although females seemed to exhibit a higher resistance to muscle fatigue than males, the contralateral motor irradiation during a 30-s unilateral changed in a similar fashion in both groups. In addition, the results show a novel result in literature as the 30-s unilateral sustained MVC induced a contralateral potentiation effect in the post-exercise MVC for the females only.

### Maximal voluntary contractions and muscle activity in the EL and NEL

The reduction of the EL MVC after the 30-s exercise indicated the existence of an induced muscle fatigue for both males and females. Yet, our results suggest that females were more fatigue-resistant than men, as previously shown [27]. Surprisingly, while no change in the muscle activity from PRE to POST, MVC was found for either group. It is possible that the mechanisms of muscle fatigue might have been of a different origin for males and females. The intramuscular pressure is lower for weaker individuals, as stronger ones have a greater occlusion of blood flow, lessening the role of muscle afferents [28, 40]. Thus, males would experience greater modifications of the neural drive and changes at the peripheral level, accounting for a greater decrement in maximal voluntary contraction than for the females. In addition, recent research [44] has shown that females recovered at a quicker rate than males after a fatiguing isometric knee extension-sustained MVC. Most likely, a lower level of central fatigue [44] and a reduced role of groups III and IV afferents onto the motorneuron pool in females compared to males could explain the lower reduction in MVC for the females.

For both groups, the normalized sEMG RMS activity of the EL knee extensors was not found to be modified, suggesting that the neural drive input to the muscles was not altered [9, 10]. Yet, a decrease in maximal force production was found. With solely EMG, it is difficult to decipher the contributions between peripheral and central factors of muscle fatigue [51]. While it is possible that the overall excitatory drive to the motor neuron pool was preserved, with a non-modification of the sEMG signal, maybe peripheral factors such modification in the performance of the contractile apparatus [10, 51] can explain the decrement of MVC.

Two main mechanisms could explain the sex difference in the NEL sEMG in the VL. First, the post-activation potentiation at the peripheral level may have led to an increased Ca^2+^ sensitivity due to regulatory light chain phosphorylation [10, 52]. Second, it is likely that a potentiation caused by the facilitation of neurotransmitters availability in the spinal cord via the Ia afferents occurred [41–44] via the crossed-extensor reflex pathways [19] or other neural mechanisms such as increased motor units recruitment, discharge rate, or synchronization [52]. Muscle fatigue and potentiation can co-exist [52].

Another possibility is that interhemispheric connections via transcallosal pathways would inhibit the motor cortex projecting to the contralateral resting limb. Both inhibitory [53] and facilitatory [54] pathways exist with fatiguing muscle contraction. As already suggested by Martin and Rattey [13], maybe the regulation of inhibitory effects could be of different magnitude for males and females. We may therefore speculate that facilitatory neural pathways [54] could partly explain the potentiation effects. Altogether, this might explain the increase in the sEMG RMS activity of the VL for the females.

The 30-s unilateral exercise was not enough to produce a reduction in the contralateral resting limb MVC. This both contrasts [5, 13, 14, 24–26, 45–49] and agrees [22, 55–57] with the literature. Whether a cross-over effect of muscle fatigue, shown as a decline in MVC of the NEL, arises or not is multifaceted. The training status [58] might play a role, but more importantly, most likely experimental procedures being carried out may explain this discrepancy. While one study showed a 13% force decline in knee extensors in males after a 100-s MVC bout [13], some other studies showed declines of 4.1% [14] and 4.9% [5] after a single bout of 100-s MVC. Yet, in the present study, the unilateral exercise was only of 30-s duration, about a third of the times of the previously referred studies. In addition, the subsequent rest period may have allowed the neuromuscular system to recover quickly enough. The supraspinal fatigue observed after a submaximal quadriceps exercise to task failure were shown to recover within less than 2 min [59]. Thus, it cannot be excluded that a part of central fatigue recovery hampered the observation of the cross-over effect of muscle fatigue. Nonetheless and interestingly, it is to be highlighted that a potentiation effect was observed in the VL muscle activity of the NEL for the females.

### Thirty-second unilateral exercise

During the 30-s exercise, females did not exhibit a force output reduction whereas men did. Surprisingly, for both males and females, the knee extensor muscle activity of the EL did not modify throughout the test, suggesting that the neuromuscular system was capable to compensate for the demand of the strenuous task. Possibly, an increase in the descending neural drive recruiting additional motor units [8, 9] and/or a compensatory mechanism of motor unit rotation might be main candidate mechanisms [60, 61]. The descending neural drive or its transmission from the central nervous system to the muscle fibers through the α-MNs at the spinal level can be modified [8, 9]. Concomitantly, the firing rate of motor units varies in a cyclic fashion—a motor unit activity substituting to one another to provide a metabolic rest for the contractile apparatus—ensuring the continuation of the task and offset the muscle fatigue [61]. As the participants were asked to perform a 30-s exercise, it is very likely that, consciously or not, they have matched the effort to its expected duration or did not reach their maximal potential at the start of the exercise [62–64].

Interestingly, males and females exhibited a similar increase in the NEL sEMG RMS activity in the present study. Possible underlying mechanisms might lie in central adjustments for the cross-transfer [2–4, 18, 65]. Adaptations seem to occur both at the spinal and supraspinal levels. At the segmental level, the amplitude of contralateral Hoffman reflex has been demonstrated to be reduced in the lower limb [66] and in the upper limb [15, 19]. Additionally, interhemispheric regulation through transcallosal pathways and therefore increase in corticospinal excitability of the contralateral side might occur [54, 67] and thus yield a bilateral corticospinal activity [15, 68, 69]. Moreover, as about 10 to 15% of the ventral corticospinal fibers do not decussate at the medulla, the role of the ipsilateral fibers to the non-exercised limb could account for the cross-transfer of muscle activity we have observed [69, 70]. Therefore, the motor irradiation observed during the 30-s exercise seemed to be independent of the sex of the participants.

### Limitations of the study

Some limitations to the study should be pointed out. First, with 9 and 11 volunteers in each group, the sample size is comparatively low. Nevertheless, several statistically significant results were observed. Furthermore, effect sizes and observed power were reported with all statistical results to alleviate this issue. Second, the brief rest period after the 30-s unilateral exercise has provided a recovery process to both limbs. As the cross-over effect seems to be dependent on the amount of unilateral activity [5], the rest period might be one reason why the MVC was not observed to decrease in the NEL. Third, another potential limitation lies in the choice of the tested limb. In the current study, the strongest limb was chosen to provide a homogeneous way of testing the participants; however, this could also have produced as a possible confounding factor since it is unknown whether the limb dominance plays a role in the cross-over effect. However, the authors are not aware of the evidence in the literature suggesting that limb laterality may affect the cross-over effect. Fourth, it cannot be excluded that our female participants did not fully activate their muscles during the 30-s exercise. Therefore, this could be a candidate argument to explain the greater fatigue resistance (lower reduction in MVC from PRE to POST) for females compared to males.

## Conclusion

The current study suggested that even though females were less prone to muscle fatigue than men, motor irradiation and increase in muscle activation were of similar magnitude in the non-exercising contralateral limb during a unilateral contraction. In addition, females exhibited signs of contralateral potentiation in the post-exercise MVC. It is probable that underlying mechanisms might differ between males and females. Yet, a similar relative intensity of muscle contraction seems to similarly influence the magnitude of cross-transfer effect in both males and females.

## Abbreviations

ANOVA: Analysis of variance
CNS: Central nervous system
EL: Exercising limb
EMG: Electromyography
MU: Motor unit
MVC: Maximal voluntary contraction
NEL: Non-exercising limb
POST: Post-30-s exercise test
PRE: Pre-30-s exercise test
RF: Rectus femoris
RMS: Root mean square
sEMG: Surface electromyography
VL: Vastus lateralis
VM: Vastus medialis
